# Diabetes mellitus – Definition, Klassifikation, Diagnose, Screening und Prävention (Update 2023)

**DOI:** 10.1007/s00508-022-02122-y

**Published:** 2023-04-20

**Authors:** Jürgen Harreiter, Michael Roden

**Affiliations:** 1 Abteilung für Endokrinologie und Stoffwechsel, Universitätsklinik für Innere Medizin III, Medizinische Universität Wien, Wien, Österreich; 2 Institut für Klinische Diabetologie, Deutsches Diabetes-Zentrum (DDZ), Leibniz-Zentrum für Diabetesforschung, Düsseldorf, Deutschland; 3 Klinik für Endokrinologie und Diabetologie, Medizinische Fakultät, Universitätsklinikum Düsseldorf, Heinrich-Heine-Universität, Düsseldorf, Deutschland; 4 Deutsches Zentrum für Diabetesforschung, DZD e. V., München-Neuherberg, Deutschland

**Keywords:** Diabetes mellitus, Hyperglykämie, Glukoseintoleranz, Abnorme Nüchternglukose, Prädiabetes, Adipositas, Screening, Prävention, Diabetes mellitus, Hyperglycemia, Glucose intolerance, Impaired fasting glucose, Prediabetes, Obesity, Screening, Prevention

## Abstract

Diabetes mellitus bezeichnet eine Gruppe von heterogenen Erkrankungen, deren gemeinsamer Befund die Erhöhung der Blutglukosekonzentration ist. Die gegenwärtige Klassifikation des Diabetes mellitus wird dargestellt und die wesentlichen Merkmale von Typ 1 und Typ 2 Diabetes werden beschrieben. Darüber hinaus werden die Kriterien für die korrekte biochemische Diagnose unter Nüchtern-Bedingungen und im oralen Glukosetoleranz-Test sowie die Anwendung des Hämoglobin A1c (HbA1c) zusammengefasst. Die zunehmende Prävalenz des Diabetes erfordert zudem gezieltes Screening zur Erkennung von Diabetes und Prädiabetes in Risikogruppen. Dies bildete die Grundlage für die frühzeitige Einleitung von Maßnahmen zur Prävention der Manifestation des Diabetes in diesen Risikogruppen und Verzögerung der Diabetesprogression.

## Abstract (en)

Diabetes mellitus comprises of a group of heterogeneous disorders, which have an increase in blood glucose concentrations in common. The current classification for diabetes mellitus is presented and the main features of type 1 and type 2 diabetes are compared. Furthermore, the criteria for the correct biochemical diagnosis during fasting and oral glucose tolerance tests as well as the use of hemoglobin A1c (HbA1c) are summarized. The increasing prevalence of diabetes requires targeted screening for detecting diabetes and prediabetes in risk groups. This forms the basis for the early initiation of measures to prevent the onset of diabetes in these risk groups and to delay the progression of diabetes.

Im Jahr 2021 wurde die weltweite Prävalenz von Diabetes mellitus bei Erwachsenen zwischen dem 20. und 79. Lebensjahr auf 537 Mio., vor allem bedingt durch Typ‑2 Diabetes mellitus, geschätzt [1]. Bis zum Jahr 2045 soll diese Zahl um 46 % auf 783 Mio. zunehmen, wobei dies in besonderen Maß strukturärmere Regionen betreffen soll (Prävalenz: 13–134 %) [1]. Für Niedriglohnländer wird zudem eine hohe Prävalenz an nicht diagnostiziertem Diabetes angenommen (24–54 %) [1]. Die in Österreich aufgrund fehlender nationaler Register geschätzte Diabetesprävalenz beträgt gemäß des Österreichischen Diabetesberichts aus dem Jahr 2017 etwa 5–7 % [2]. Da bereits Vorstufen des Diabetes („Prädiabetes“) mit erhöhtem Risiko für vaskuläre Erkrankungen (koronare Herzkrankheit, Schlaganfall) und allgemeine Mortalität assoziiert sind, sind effiziente Strategien zu Früherkennung und Prävention von Prädiabetes und Typ 2 Diabetes erforderlich [3].

## Definition

Diabetes mellitus bezeichnet eine Gruppe von Stoffwechselerkrankungen, deren gemeinsamer Befund die Erhöhung des Blutglukosespiegels, die Hyperglykämie, ist. Schwere Hyperglykämie führt von klassischen Symptomen wie Polyurie, Polydipsie, Müdigkeit und Leistungsabfall, unerklärbarer Gewichtsverlust über Sehstörungen und Infektanfälligkeit bis hin zu Ketoazidose oder nicht-ketoazidotischem, hyperosmolaren Syndrom mit Gefahr des Komas. Chronische Hyperglykämie bewirkt desweitern Störungen der Sekretion und/oder Wirkung von Insulin und ist mit Langzeitschäden und Funktionsstörungen verschiedener Gewebe und Organe (Augen, Nieren, Nerven, Herz und Blutgefäße) sowie Krebserkrankungen assoziiert.

## Klassifikation

Die Klassifikation des Diabetes mellitus erfolgt in 4 Typen [4, 5].

### Typ 1 Diabetes

Störung der Insulinsekretion durch überwiegend immunologisch vermittelte Zerstörung der pankreatischen Betazellen mit meist absolutem Insulinmangel. LADA (latenter autoimmuner Diabetes der Erwachsenen) bezeichnet einen autoimmun-bedingten Diabetes mellitus, der durch das Auftreten im Erwachsenenalter und den langsameren Verlust der Insulinsekretion gekennzeichnet ist, dem Typ 1 Diabetes zugeordnet wird und keinen eigenständigen Subtyp darstellt. Das Vorhandensein von Diabetes-assoziierten (auch: Inselzell‑) Autoantikörpern ist ein starker Prädiktor für die Entwicklung eines Typ 1 Diabetes. Dabei scheinen Alter bei Feststellung, Titerhöhe sowie Anzahl und Spezifität der Autoantikörper mit der Progression des Typ 1 Diabetes assoziiert zu sein [4].

### Typ 2 Diabetes

Verminderung der Insulinwirkung (Insulinresistenz) mit fortschreitendem Verlust der Betazellfunktion, bei zunächst häufig relativem Insulinmangel und typischerweise Störung der glukoseabhängigen Insulinsekretion. Die Funktionsstörungen sind in unterschiedlicher Ausprägung schon lange vor der klinischen Manifestation des Diabetes allein oder im Rahmen eines metabolischen Syndroms mit erhöhtem Risiko für makrovaskuläre Folgen vorhanden. Tab. 1 listet Hinweise zur klinischen Differentialdiagnose zum Typ 1 Diabetes auf.

**Table 1:** 

Kriterium^a^	Typ 1 Diabetes	Typ 2 Diabetes
Häufigkeit	Selten, < 10 % der Diabetes-Fälle	Häufig, > 90 % der Diabetes-Fälle
Manifestationsalter	Meist in Kindheit oder Jugend (Ausnahme: LADA)	Meist im höheren Alter, zunehmend frühere Manifestation
Körpergewicht	Meist normalgewichtig	Meist übergewichtig, adipös
Symptome	Häufig	Seltener
Neigung zur diabetischen Ketoazidose (DKA)	Ausgeprägt	Fehlend oder nur gering
Familiäre Häufung	Gering	Typisch
Plasma C‑Peptid	Meist niedrig bis fehlend	Meist normal bis erhöht
Diabetes-assoziierte Antikörper	85–95 % + (GAD, ICA, IA‑2, IAA, ZnT8)	Keine
HLA-Assoziation	+ (HLA-DR/DQ)	Keine
Insulintherapie	Sofort erforderlich	Oft erst nach längerem Verlauf und nach erfolgloser Lebensstilmodifikation und oraler Diabetestherapie erforderlich

^a^Symptome, Klinik und Verlauf beider Diabetestypen weisen eine hohe Variabilität auf, die eine Differentialdiagnose im Einzelfall erschweren kann. Bei unklarem Diabetestyp oder untypischer Klinik sollte immer auch an andere seltene Diabetesformen (z. B. MODY) gedacht werden

Rezente Studien schlagen eine weitere Klassifizierung des Typ 2 Diabetes in 5 „Cluster“ (Subtypen, Endotypen) vor, die Unterschiede im Ausmaß der Insulinresistenz und Betazellfunktion sowie Risiken für Diabetes-bedingte Komplikationen aufweisen sollen [6, 7]. Eine solche Klassifikation könnte zukünftig als Basis einer neuen Typisierung des Diabetes dienen und eine stratifizierte bzw. präzisere Prävention und Therapie ermöglichen. Dies erfordert allerdings weitere Studien, sodass derzeit eine solche Klassifizierung für die klinische Praxis noch nicht empfohlen werden kann.

### Andere spezifische Diabetes-Typen

Erkrankungen des exokrinen Pankreas (z. B. Pankreatitis, Traumen, Operationen, Tumoren, Hämochromatose, zystische Fibrose), endokriner Organe (z. B. Cushing-Syndrom, Akromegalie), medikamentös-chemisch (z. B. Glukokortikoide, α‑Interferon, Posttransplantations-Diabetes, HAART bei HIV/AIDS), genetische Defekte der Insulinsekretion (z. B. Formen des Maturity Onset Diabetes of the Young [MODY]) und der Insulinwirkung (z. B. Lipoatropher Diabetes), andere genetische Syndrome (z. B. Down‑, Klinefelter‑, Turner-Syndrome), Infektionen (z. B. kongenitale Röteln) und seltene Formen des autoimmun-vermittelten Diabetes (z. B. „Stiff-man“-Syndrom). Details finden sich in der Leitlinie „Andere spezifische Diabetesformen und exokrine Pankreasinsuffizienz“.

### Gestationsdiabetes (GDM)

Erstmals im zweiten oder dritten Schwangerschaftstrimester aufgetretene bzw. diagnostizierte Glukosetoleranzstörung. Voraussetzung ist, dass außerhalb der Schwangerschaft kein Diabetes mellitus bestanden hat. (siehe: Leitlinie „Gestationsdiabetes“).

Aufgrund einer oft unklaren Vorgeschichte ist eine Unterscheidung zwischen den einzelnen Diabetestypen zum Zeitpunkt der Diagnosestellung vor der notwendigen ausführlichen Anamnese und dem Eintreffen aller erforderlichen Befunde nicht immer möglich. Insulinabhängigkeit stellt keine Klassifikation dar.

## Diagnose

Die Diagnose des Diabetes erfolgt anhand von Nüchtern-Glukose, Gelegenheitsglukose, oralem Glukosetoleranz-Test (OGTT) oder Hämoglobin A1c (HbA1c). Die Hyperglykämie entwickelt sich kontinuierlich und die Störungen von Nüchtern- und postprandialer Glykämie weisen unterschiedliche Zeitverläufe auf. Die etablierten Diagnosegrenzwerte der jeweiligen Parameter sind daher nicht immer in kompletter Übereinstimmung bei der Identifizierung von Menschen mit Diabetes, desweitern unterliegen alle Tests einer Variabilität, so dass eine zeitnahe Testwiederholung oder Bestätigung eines Testresultates durch einen anderen Test – außer bei Vorliegen klassischer klinischer Symptome – immer erforderlich sind.

### Nüchtern-Glukose, Gelegenheitsglukose und OGTT

Die Diagnose wird unabhängig von Alter und Geschlecht durch Messung mehrfach erhöhter Blut-Glukosewerte an mindestens zwei verschiedenen Tagen gestellt (Tab. 2). Bei klinischem Verdacht und widersprüchlichen Ergebnissen wird die Diagnose mittels OGTT gestellt. Als „normal“ gelten derzeit Nüchtern-Glukose-Werte im venösen Plasma von < 100 mg/dl (< 5,6 mmol/l) bzw. postprandiale Werte < 140 mg/dl (< 7,8 mmol/l). Niedrigere Werte schließen das Vorliegen von einer Glukosestoffwechselstörung oder Folgeschäden aber nicht aus. Die Grundlage für die Wahl der Grenzwerte liegt in der überwiegend kontinuierlichen Beziehung zwischen höheren Blutglukose-Werten (nüchtern und 2 h nach oraler Glukosebelastung) und der Zunahme des Risikos für Folgeschäden.

**Table 2:** 

	Manifester Diabetes mellitus	Erhöhtes Diabetes-Risiko (Prädiabetes)^a^
Nicht-Nüchtern, Gelegenheitsglukose („Random-Glucose“, venös od. kapillär)	≥ 200 mg/dl (11,1 mmol/l) an 2 Tagen^b^ ODER ≥ 200 mg/dl (11,1 mmol/l) + klassische Symptome^c^	–
Nüchtern-Glukose (venöses Plasma)	≥ 126 mg/dl (7,0 mmol/l) an 2 Tagen^b^	≥ 100 mg/dl (5,6 mmol/l), aber < 126 mg/dl (7,0 mmol/l) (Abnorme Nüchternglukose, „impaired fasting glucose“, IFG)
2‑h-Glukose nach 75 g OGTT (venöses Plasma)	≥ 200 mg/dl (11,1 mmol/l) an 2 Tagen^b^	Glukose ≥ 140 mg/dl (7,8 mmol/l), aber < 200 mg/dl (11,1 mmol/l) (Gestörte Glukosetoleranz, „impaired glucose tolerance“, IGT)
HbA1c	≥ 6,5 % (48 mmol/mol) an 2 Tagen^b^	≥ 5,7 % (39 mmol/mol), aber < 6,5 % (48 mmol/mol)^d^

^a^Ein erhöhtes Diabetes-Risiko kann auch ohne Nachweis von Störungen der Glykämie bestehen und lässt sich mittels definierter Risiko-Tests erheben (siehe unter: Prävention)

^b^Sind 2 unterschiedliche Tests positiv, ist die Diagnose Diabetes gegeben, so dass auf die Testwiederholung verzichtet werden kann. Ergeben unterschiedliche Tests unterschiedliche Ergebnisse, dann ist der Test mit erhöhtem Ergebnis zu wiederholen

^c^Bei Vorliegen von Hyperglykämie und klassischen Symptomen ist die Diagnose ohne Testwiederholung gegeben, da z. B. bei Erstmanifestation des Typ 1 Diabetes das HbA1c normal sein kann

^d^Weiterführende Diagnostik mittels Nüchtern-Glukose oder OGTT ist erforderlich

Für die Diagnose des GDM gelten andere als in Tab. 2 gelistete Kriterien (siehe ÖDG-Leitlinien zu Gestationsdiabetes für detaillierte Informationen) [8]. Die Durchführung eines 75 g OGTT wird in der 24–28. Schwangerschaftswoche bei allen Frauen ohne bereits vorbestehenden Diabetes mellitus empfohlen. Die Grenzwerte lauten nüchtern < 92 mg/dl (5,1 mmol/l), nach 1 h < 180 mg/dl (10,0 mmol/l) und nach 2 h < 153 mg/dl (8,5 mmol/l) [8]. Bereits ab einem erhöhten Plasmaglukosewert wird die Diagnose Gestationsdiabetes gestellt.

Voraussetzungen zur Glukosebestimmung sind:

- ausschließlicher Einsatz qualitätsgesicherter Maßnahmen oder Tests
- vorzugsweise Bestimmung im venösen Plasma (Zusatz von Lithium-Heparin oder besser EDTA + Natrium-Fluorid). Serumproben sind nur zu verwenden, wenn ein Glykolysehemmstoff zugesetzt wurde
- keine Bestimmung mit Blutglukosemessgeräten, die zur Selbstkontrolle verwendet werden
- „Nüchtern“ bedeutet eine Zeit von ≥ 8 h ohne jegliche Kalorienaufnahme
- Bei der Durchführung ist auf die mögliche Verfälschung der Diagnose durch interkurrente Erkrankungen (z. B. Infektionen, Dehydratation, gastrointestinale Krankheiten) oder Medikamenten-Einnahme (z. B. Glukokortikoide) zu achten
- Bei Situationen/Erkrankungen mit erhöhtem Erythrozytenumsatz (z. B. Schwangerschaft, Hämodialyse, Bluttransfusion, großer Blutverlust, Sichelzellanämie, Thalassämie, hereditäre Sphärozytose) sollte nur die Plasmaglukose-Konzentration zur Diagnose des Diabetes mellitus herangezogen werden, da der HbA1c-Wert in diesen Fällen falsch niedrig sein kann.

### HbA1c

Mit den ÖDG-Leitlinien 2012 wurden auch erhöhte HbA1c-Werte in die Standardkriterien zur Diagnose des Diabetes mellitus übernommen [9, 10]. Demgemäß kann ein Diabetes mellitus anhand der HbA1c-Grenzwerte ≥ 6,5 % (48 mmol/mol) diagnostiziert werden (Tab. 2). Grundlage dafür ist die Zunahme des Risikos für diabetische Retinopathie ab HbA1c-Werten von > 6,5 % (48 mmol/mol) [10, 11]. Für HbA1c-Werte von 5,7 % (39 mmol/mol) bis einschließlich 6,4 % ist ein erhöhtes Diabetes-Risiko anzunehmen, so dass in diesem Fall eine Abklärung mittels Nüchtern-Glukose und OGTT empfohlen wird. Vergleichbare HbA1c-Schwellenwerte für andere mikrovaskuläre oder makrovaskuläre Diabetesfolgen sind bisher nicht etabliert [11]. Diabetes mellitus kann bei erwachsenen Personen, Kindern und Jugendlichen auch auf Basis eines erhöhten HbA1c-Wertes diagnostiziert werden [4, 12]. Die Vorteile der Messung des HbA1c Wertes liegen in der höheren präanalytischen Stabilität und geringerer täglicher Varianz [4]. Nachteile sind die geringere Sensitivität, höhere Kosten (somit weltweit nicht überall verfügbar) und geringeren Korrelation zwischen HbA1c und durchschnittlichen Blutglukosewerten. Die Bestimmung des HbA1c ist eine indirekte Messung der durchschnittlichen Blutglukosewerte über mehrere Wochen hinweg und kann durch Einflussfaktoren wie Alter, Ethnizität und Anämie/Hämoglobinopathie von den tatsächlich gemessenen Glukosewerten abweichen [4]. Von besonderer Bedeutung ist die eingeschränkte Aussagekraft des HbA1c-Wertes unter den folgenden Umständen, die den Einsatz des HbA1c zur Diagnose des Diabetes mellitus ausschließen sollten:

- Veränderungen des Hämoglobins (Hb): z. B. angeborene Hämoglobinopathien (HbS, HbE, HbF, HbC, HbD); Hb-Modifikationen bei Urämie (karbamyliertes Hb) oder Azetylsalizylsäure in hohen Dosen (azetyliertes Hb)
- Veränderung der Erythrozyten-Lebensdauer (Umsatz, Turnover): z. B. verlangsamter Umsatz bei Eisenmangel- und Vitamin-B12-Mangel-Anämien oder Niereninsuffizienz erhöht das HbA1c; beschleunigter Umsatz bei hämolytischen Anämien oder chronischen Leber-Erkrankungen senkt das HbA1c
- Hemmung der Glykierung: z. B. Dauertherapie mit Vitamin C oder Vitamin E
- Schwangerschaft: 2. und 3. Trimester
- Alter: bei identen Glukosewerten nimmt das HbA1c mit dem Alter zu
- Ethnizität: z. B. höhere HbA1c-Werte bei Afroamerikanern und Südasiaten als bei nicht hispanischen Weißen
- Labortechnischen Probleme: Unerklärliche Abweichungen zwischen HbA1c und Plasmaglukose sollten an labortechnische Probleme bei der HbA1c-Bestimmung denken lassen (z. B.: Assayinterferenz). Nähere Informationen zu Faktoren, die mit HbA1c-Ergebnissen interferieren können und Assay-Interferenzen sind im Internet unter www.ngsp.org (http://www.ngsp.org/factors.asp, http://www.ngsp.org/interf.asp) nachzulesen

Zur besseren Vergleichbarkeit der Methoden zur Bestimmung des HbA1c sollen ausschließlich Methoden verwendet werden, die nach dem neuen Standard der International Federation of Clinical Chemistry (IFCC) referenziert sind [9, 13]. Diese Werte sollen, um Verwechslungen zu vermeiden, nach dem IFCC-Standard in mmol/mol ausgegeben werden. Die Umrechnung in den HbA1c-Wert in Prozent nach dem National Glycohemoglobin Standardization Program (NGSP) bzw. dem Diabetes Control and Complications Trial (DCCT) ist wie folgt:

1 $$HbA1c\,\textit in\,\textit{Prozent}=(0{,}09148*HbA1c\,\textit in\,\textit mmol/mol)+2{,}152$$

Ein DCCT HbA1c-Wert von 6,5 % entspricht somit einem IFCC-HbA1c von 48 mmol/mol.

## Oraler Glukosetoleranztests (OGTT) nach WHO-Richtlinien

- Indikationen:
  Risikogruppen (siehe unten), ältere Menschen (aber nicht routinemäßig), gestörte Nüchternglukose, Schwangerschaft in der 24–28. Schwangerschaftswoche (siehe auch: Leitlinie Gestationsdiabetes)
- Durchführung:
  ≥ 3 Tage kohlenhydratreiche (≥ 150 g/Tag) Ernährung 10–16 h Nahrungs- und Alkohol-Karenz vor dem Test

Testung am Morgen im Liegen/Sitzen (kein Rauchen vor/während des Tests, keine übermäßige körperliche Aktivität).

### Glukosebestimmung (Zeitpunkt 0 min)

Trinken von 75 g Glukose (oder äquivalente Menge Stärke) in 250–350 ml Wasser (Kinder: 1,75 g/kg bis maximal 75 g Glukose) innerhalb 5 min.

Glukosebestimmung zum Zeitpunkt 60 min nach Glukoseaufnahme: nur bei Abklärung von GDM notwendig.

### Glukosebestimmung (Zeitpunkt 120 min nach Glukoseaufnahme)

- Kontraindikationen:
  interkurrente Erkrankungen, St. p. Magen-Darm-Resektion/bariatrische Operation, Resorptionsstörungen, nachgewiesener Diabetes mellitus
- Einflussfaktoren:
  Längeres Fasten, Kohlenhydrat-Mangelernährung können auch bei Gesunden zur pathologischen Glukosetoleranz führen. Eine Reihe von Medikamenten, wie z. B. Glukokortikoide, Adrenalin (Epinephrin), Phenytoin und Furosemid können die Glukosetoleranz verschlechtern

## Screening

Personen mit erhöhtem Diabetesrisiko sollten durch systematisches Screening auf Prädiabetes und Typ 2 Diabetes untersucht werden. Die Risikofaktoren vor allem für Typ 2 Diabetes umfassen unter anderem einen Mangel an körperlicher Aktivität und unausgewogene hyperkalorische Ernährung, die häufig die Basis für Übergewicht und Adipositas und in weiterer Folge Hyperlipidämie, arterielle Hypertonie, nicht-alkoholische Fettlebererkrankungen (nonalcoholic fatty liver disease, NAFLD) [14] bilden (Tab. 3). Weitere Risikofaktoren stellen eine genetische Prädisposition bzw. positive Familienanamnese, eine gewisse ethnische Herkunft, zunehmendes Lebensalter sowie Sexualhormonstörungen und Gestationsdiabetes dar [15]. Als Risikofaktor für Typ 1 Diabetes gelten Diabetes-assoziierte Antikörper, wobei das Vorliegen von 2 oder mehr dieser Autoantikörper auf ein > 80%iges Risiko für Typ 1 Diabetes innerhalb von 15 Jahren hinweist [4]. Zystische Fibrose und Zustand nach Transplantation von Organen sind ebenso Risikofaktoren für die Entstehung einer Hyperglykämie.

**Table 3:** 

*1. Ein Hyperglykämie-Screening sollte bei Vorliegen folgender Risikofaktoren erfolgen:* BMI ≥ 25 kg/m^2^ (bei asiatischer Herkunft 23 kg/m^2^) Positive Familienanamnese bei erstgradigen Verwandten Ethnizität mit erhöhtem Diabetesrisiko (asiatische, afrikanische, lateinamerikanische Herkunft) Vaskuläre Erkrankungen Arterielle Hypertonie (≥ 140/90 mm Hg oder antihypertensive Therapie) HDL-Cholesterin < 35 mg/dl und/oder Triglyzeride > 250 mg/dl Polyzystisches Ovarsyndrom (PCOS) Hypogonadismus Körperliche Inaktivität Acanthosis nigricans NAFLD^a^ Chronischer Tabakkonsum^b^
*2. Bei bekanntem Prädiabetes sollte ein jährliches Screening erfolgen*
*3. Bei Zustand nach GDM sollte zumindest alle 3 Jahre ein Screening erfolgen*^c^
*4. HIV-positive Personen*
*5. Bei allen anderen Personen sollte ein Screening ab einem Alter von 35 Jahren erfolgen*
*6. Bei unauffälligen Screening-Resultaten sollte ein weiteres Screening alle 3 Jahre erfolgen. Engmaschigere Kontrollen sollten den Screening-Ergebnissen und Risikofaktoren entsprechend geplant werden*

^a^Umfasst einfache Fettleber (Steatosis hepatis oder nicht-alkoholische Fettleber, NAFL), nicht-alkoholische Steatohepatitis (NASH), „kryptogene“ Formen der Leberfibrose, -zirrhose und des hepatozellulären Karzinoms [14]

^b^Chronischer Tabakkonsum ist mit erhöhtem T2DM Risiko assoziiert [16]

^c^Spezielle Risikofaktoren und Screening für GDM (siehe Leitlinie: „Gestationsdiabetes“)

Vor Durchführung von Labortests empfehlen nationale Diabetesorganisationen wie die ADA oder die DDG die Durchführung von Diabetes-Screening-Tests zur besseren Risikobewertung (z. B. FINDRISK, ADA Diabetes Risk Test, Deutscher Diabetesrisikotest). Bei Vorliegen eines der oben genannten Risikofaktoren sollte eine Testwiederholung in einem minimalen Intervall von 3 Jahren, bei Prädiabetes jährlich stattfinden.

Generell sollte ein Screening auf Typ 1 Diabetes mittels Diabetes-assoziierter Autoantikörper in der Allgemeinbevölkerung nicht durchgeführt werden, sondern wird nur bei erstgradigen Verwandten mit Typ 1 Diabetes empfohlen.

Bei Verdacht auf das Vorliegen eines monogenetischen Diabetes mellitus ist eine unmittelbare molekulargenetische Testung zu empfehlen. Dies sollte vor allem bei Auftreten von Hyperglykämie in den ersten 6 Lebensmonaten oder bei in mehreren Generationen auftretendem Diabetes mellitus mit nicht zu Typ 1 oder Typ 2 Diabetes passenden Symptomen erfolgen. Ein darauf spezialisiertes Zentrum sollte in die Versorgung dieser Personen zur Abschätzung der Signifikanz der Mutation, zur fachgerechten genetischen Beratung und Therapieplanung einbezogen werden [4]. Die Aufklärung des Betroffenen und ein genetisches Beratungsgespräch müssen entsprechend den Richtlinien des Gentechnikgesetztes erfolgen (siehe auch Leitlinie „Andere spezifische Diabetesformen und exokrine Pankreasinsuffizienz“).

Bei Zustand nach Transplantation eines Organs und dementsprechend erforderlicher immunsuppressiver Therapie sowie bei Zystischer Fibrose ist das Screening der Wahl ein OGTT [4]. Bei Zystischer Fibrose sollte der OGTT jährlich, bei Zustand nach Transplantation nach Stabilisierung der immunsuppressiven Therapie durchgeführt werden.

Bei asymptomatischen Kindern und Jugendlichen sollte ebenfalls ein Typ 2 Diabetesscreening erfolgen, wenn eine Adipositas (BMI > 95. Perzentile, geschlechts- und altersadjustiert) oder ein Übergewicht (BMI > 85. Perzentile) und zusätzlich ein oder mehrere Risikofaktoren wie mütterlicher GDM in der Schwangerschaft des Kindes oder Typ 2 Diabetes bei Verwandten 1. bis 2. Grades, Hinweis auf Insulinresistenz oder mit ihr assoziierte Veränderungen oder Ethnizität mit erhöhtem Risiko vorliegen [4].

## Prävention

### Prävention des Typ 2 Diabetes

Zahlreiche prospektive Studien zur Prävention des Typ 2 Diabetes, unter anderem die Diabetes Prevention Study (DPS) und das Diabetes Prevention Program (DPP), zeigten, dass Veränderung des Lebensstils oder medikamentöse Maßnahmen eine Reduktion des Risikos der Manifestation von Typ 2 Diabetes mellitus ermöglichen (Tab. 4 und 5; [17, 18]). Mittels Lebensstilmodifikation konnte das Diabetesrisiko um 39 % und mit medikamentösen Interventionen um 36 % reduziert werden, jedoch konnte eine langfristige Risikoreduktion (28 %, durchschnittliche Beobachtungszeit 7,2 Jahre) nur mit Lebensstilveränderung beobachtet werden [17, 18]. Nach medikamentöser Intervention war keine nachhaltige Reduktion des Diabetesrisikos zu erkennen [17]. Auch die Kosten-Nutzen-Rechnung zeigt deutlich die längerfristigen positiven Effekte von Lebensstilintervention auf. In einer britischen Berechnung zur Kosteneffektivität waren sowohl Lebensstilmaßnahmen mit niedriger als auch hoher Intensität und medikamentöse Intervention mit Metformin im Vergleich zu keiner Intervention bei Anwendung an Personen mit IFG, IGT oder erhöhtem HbA1c (Prädiabetes) kosteneffektiv [19]. Bislang zeigte nur die chinesische DaQing Diabetes Prevention Study eine Reduktion der kardiovaskulären und allgemeinen Mortalität bei Frauen mit IGT [20]. In der Interventionsgruppe wurde nach einem 6‑jährigen Lebensstilinterventionsprogramm die kardiovaskuläre Mortalität um 41 % (*n* = 51/430;12 % vs. *N* = 27/138; 20 %; HR 0,59, 0,36–0,96) und die allgemeine Mortalität um 29 % (*n* = 121/430; 28 % vs. *N* = 53/138;38 %, HR 0,71, 0,51–0,99) im Follow-up nach > 20 Jahren gesenkt. Eine Follow up-Analyse der Da Qing Diabetes Prevention Study nach 30 Jahren zeigte eine Risikoreduktion für Typ 2 Diabetes um 39 % durch Lebensstil- und Gesundheitsverhaltensänderung [21]. Neuere Daten aus der Da Qing Diabetes Prevention Study weisen darauf hin, dass eine Regression von IGT zu normaler Glukosetoleranz oder eine Verhinderung einer Progression zu Typ 2 Diabetes nach 6 Jahren Intervention auch zu einem niedrigeren Risiko für kardiovaskuläre und mikrovaskuläre Ereignisse nach über 30 Jahren Follow up führt [22]. Auch in der Diabetes Prevention Study wurde gezeigt, dass das Erreichen von Normoglykämie während der Intervention mit einem niedrigeren Risiko für Diabetes und mikrovaskulären Komplikationen in Zusammenhang steht [23].

**Table 4:** 

Studie	Studienarme	Teilnehmer	Beobachtungsdauer	Diabetesrisikoreduktion	Referenz
DPP	Ernährung + Bewegung, Metformin, Plazebo	3234 mit IFG oder IGT	3 Jahre	58 %	[28]
DPS	Ernährung + Bewegung, Kontrolle	552 Männer und Frauen mit IGT	Mittelwert 3,2 Jahre	58 %	[29]
Da Qing	Ernährung, Bewegung, Ernährung + Bewegung	577 Männer und Frauen mit IGT	6 Jahre	Bewegung: 37 % Ernährung: 33 %, Ernährung + Bewegung: 32 %	[30]
IDPP	Ernährung + Bewegung, Metformin, Ernährung, Bewegung + Metformin, Kontrolle	531 Männer und Frauen mit IGT	Median 30 Monate	Ernährung + Bewegung: 28,5 %	[31]
SLIM	Ernährung + Bewegung, Kontrolle	147 Männer und Frauen mit IGT	3 Jahre	58 %	[32]
EDIPS	Ernährung + Bewegung, Kontrolle	102 Männer und Frauen mit IGT	3,1 Jahre	55 %	[33]
Zensharen	Ernährung + Bewegung, Kontrolle	641 Männer und Frauen mit IFG	3 Jahre	44 %	[34]
JDPP	Ernährung + Bewegung, Kontrolle	304 Männer und Frauen mit IGT	3 Jahre	47 %	[35]

**Table 5:** 

Arzneistoff	Studie	Studienarme	Teilnehmer	Beobachtungsdauer	Diabetesrisikoreduktion	Referenz
Metformin	US DPP	Lebensstil, Metformin, Plazebo	3234 Männer und Frauen mit IFG oder IGT	3 Jahre	31 %	[28]
Metformin	Indian DPP	Lebensstil, Metformin, Plazebo, Metformin und Lebensstil	531 Männer und Frauen mit IGT	3 Jahre	Metformin: 26,4 % Metformin und Lebensstil: 28,2 %	[31]
Metformin + Rosiglitazon	CANOE	Metformin und Rosiglitazon, Plazebo	207 Männer und Frauen mit IGT	Median 3,9 Jahre	66 %	[50]
Glimepirid	NANSY	Glimepirid, Plazebo	274 Männer und Frauen mit IGT	3,71 Jahre	Nicht signifikant	[51]
Pioglitazon	ACT NOW	Pioglitazon, Plazebo	602 Männer und Frauen mit IGT	Median 2,4 Jahre	72 %	[52]
Rosiglitazon	DREAM	Rosiglitazon, Ramipril, Plazebo, Rosiglitazon und Ramipril	5269 Männer und Frauen mit IFG/IGT	Median 3 Jahre	60 %	[53]
Ramipril	DREAM	Rosiglitazon, Ramipril, Plazebo, Rosiglitazon und Ramipril	5269 Männer und Frauen mit IFG/IGT	Median 3 Jahre	Nicht signifikant	[54]
Acarbose	STOP-NIDDM	Acarbose, Plazebo	714 Männer und Frauen mit IGT	3,3 Jahre	25 %	[55]
Voglibose	Kawamori et al	Voglibose, Plazebo	1780 Frauen und Männern mit IGT	4 Jahre	54 %	[56]
Nateglinid	NAVIGATOR	Nateglinid, Valsartan, Plazebo, Nateglinid und Valsartan	9306 Männer und Frauen mit IFG und mindestens 1 kardiovaskulärem Risikofaktor	Median 5 Jahre	Nicht signifikant	[57]
Valsartan	NAVIGATOR	Nateglinid, Valsartan, Plazebo, Nateglinid und Valsartan	9306 Männer und Frauen mit IFG und mindestens einem kardiovaskulären Risikofaktor	Median 5 Jahre	14 %	[58]
Insulin Glargin	ORIGIN	Insulin Glargin, Standardversorgung	12,537 Frauen und Männer mit kardiovaskulären Risikofaktoren und IFG, IGT oder T2DM (1452 ohne Diabetes mellitus)	Median 6,2 Jahre	28 %	[59]
Orlistat	XENDOS	Orlistat, Plazebo	3305 Männer und Frauen mit BMI ≥ 30 kg/m^2^	4 Jahre	37 %	[60]
Bezafibrat	BIP	Bezafibrat, Plazebo	339 adipöse Frauen und Männer	Median 6,3 Jahre	32 %	[61]
Liraglutid	SCALE	Liraglutid, Plazebo	2254 Frauen und Männer mit BMI ≥ 30 kg/m^2^ oder ≥ 27 kg/m^2^ mit Komorbiditäten	3 Jahre	79 %	[62]
Testosteron	T4DM	Testosteron undecanoate, Plazebo	1007 übergewichtigeMänner mit IGT oder neu diagnostiziertem T2DM	2 Jahre	41 %	[63]

Da sowohl Rauchen als auch Passivrauchen die Inzidenz für Diabetes erhöht, trägt eine rauchfreie Umgebung unmittelbar zur Diabetesprävention bei [24]. Ein Rauchstopp kann zwar über die mögliche Gewichtszunahme das Diabetesrisiko mittelfristig erhöhen, senkt aber gleichzeitig die erhöhte Mortalität um nahezu 50 %. Details siehe Leitlinie „Rauchen, erhitzte Tabakprodukte, Alkohol und Diabetes“ [24]. Schlafmangel und schlechte Schlafqualität können zu Insulinresistenz und Hyperglykämie führen [25].

Bezüglich der bariatrischen (metabolischen) Chirurgie, ergab eine Metaanalyse von Studien mit insgesamt fast 95.000 Betroffenen eine Diabetesremission von > 70 % [26]. Eine weitere Metaanalyse mit 39 prospektiven und retrospektiven Kohortenstudien zeigte nach bariatrischer Operation Risikoreduktionen bei kardiovaskulären Ereignissen und Mortalität (Reduktion: Myokardinfarkt 42 %, Schlaganfall 36 %, Herzinsuffizienz 50 %, kardiovaskuläre Mortalität 41 %, Gesamt-Mortalität 45 %) [27].

Aufgrund dieser Daten erscheint es sinnvoll, mit Personen mit erhöhtem Typ 2 Diabetes Risiko Maßnahmen (Änderung des Essverhaltens, regelmäßige körperliche Aktivität) zu vereinbaren, die bei Übergewicht und Adipositas zu langfristiger Reduktion des Körpergewichts um mindestens 5–10 % führen (siehe Leitlinie „Körperliche Aktivität und Training in der Prävention und Therapie des Typ 2 Diabetes mellitus“). Um mehr Personen an einem Präventionsprogramm teilhaben zu lassen, sollten zusätzliche Angebote mithilfe neuer Technologien, z. B. webbasierter, virtueller oder mobiltelefongestützter Programme, neben den traditionellen gecoachten Programmen geschaffen werden [36]. Eine rezente Metaanalyse ergab, dass durch technologiebasierte Präventionsprogramme signifikant Gewicht reduziert und auch die glykämische Kontrolle verbessert werden können [37].

Da bereits bei Prädiabetes häufig ein erhöhtes kardiovaskuläres Risiko und Komorbiditäten des Diabetes mellitus wie Dyslipidämie oder arterielle Hypertonie vorliegen, sollten alle modifizierbaren Risikofaktoren regelmäßig kontrolliert werden [36] (siehe Leitlinien „Lipide: Diagnostik und Therapie bei Diabetes“ sowie „Antihypertensive Therapie bei Diabetes mellitus“).

### Ernährung

Die Ernährung sollte ausgewogen, ballaststoffreich und auf gesunder Mischkost basieren [36]. Eine Reduktion der Aufnahme von raffinierten Kohlenhydraten und Nahrungsmittel mit Zusatz von „Zucker“ (überwiegend Saccharose) wird empfohlen [36]. Kohlenhydrate sollten stattdessen vornehmlich aus Gemüse, Hülsenfrüchten, Obst, Milch und Vollkornprodukten bezogen werden. Vom Konsum von Getränken mit Zuckerzusatz („Softdrinks“) und prozessierten „low-fat“ Produkten mit hohem Anteil an raffiniertem Zucker wird abgeraten [36]. Die verringerte Aufnahme von gesättigten Fettsäuren und Transfettsäuren wird empfohlen. Diese sollten durch ein- oder mehrfach ungesättigte Fettsäuren ersetzt werden. Eine Kalorienreduktion sollte jedenfalls angestrebt werden und um dies zu erreichen sollte eine individualisierte Ernährungsberatung stattfinden. Um eine kontinuierliche Gewichtsreduktion zu erreichen sollte der Tagesenergiebedarf bei derzeitigem Gewicht errechnet werden und davon 500 bis 1000 Kalorien abgezogen werden. Metaanalysen zeigen, dass eher die Qualität der aufgenommenen Lipide wichtig ist und nicht die Gesamtmenge an Fett. Mediterrane Ernährung (reichlich einfach gesättigte Fettsäuren), vegetarische oder DASH (Dietary Approaches to Stop Hypertension) Diät sind mit einem niedrigeren Risiko für Entstehung eines Typ 2 Diabetes vergesellschaftet [36, 38–42]. Spezifische Nahrungsmittel (Nüsse [43, 44], Beeren [45], Joghurt [44, 46], Zimt [47], Kaffee [44]; Tee; [44]) sind in Studien mit niedrigerem Diabetesrisiko assoziiert, wohingegen rotes Fleisch und mit Saccharose angereicherte Getränke [44] das Diabetesrisiko erhöhen (für weitere Informationen siehe Leitlinie Ernährungsempfehlungen bei Diabetes mellitus).

### Körperliche Aktivität

Regelmäßige moderate körperliche Aktivität (mind. 30 min/Tag, 5 ×/Woche bei moderater Intensität, 2 × muskelkräftigendes Training/Woche) werden bei erhöhtem Diabetesrisiko und manifestem Typ 2 Diabetes empfohlen. Durch moderate körperliche Aktivität verbessert sich die Insulinsensitivität und verringert sich das abdominale Fett. Durch Aktivitätsphasen unterbrochenes längeres Sitzen führte in Studien zu geringeren postprandialen Glukosewerten [36] (für weitere Informationen siehe Leitlinie Körperliche Aktivität und Training in der Prävention und Therapie des Typ 2 Diabetes mellitus).

### Medikamente

Bisher zeigten sich Metformin, Alpha-Glukosidasehemmer, Orlistat, Thiazolidindione (Glitazone), Insulin Glargin, Glucagon-like Peptide 1 (GLP1)-Rezeptoragonisten und Testosteron effektiv in der Diabetesprävention (Tab. 5), wenngleich eine Lebensstilintervention langfristig immer noch effektiver war [36]. In zwei Metaanalysen wurde eine Reduktion des Diabetesrisikos durch ACE Hemmer und Sartane um circa 25 % und eine Steigerung des Risikos durch Statine um etwa 10 % beschrieben [48, 49]. Metformin ist das am besten untersuchte Medikament hinsichtlich Effektivität, Langzeitsicherheit und Kosteneffizienz. Bei Prädiabetes oder früherem Gestationsdiabetes, Adipositas mit BMI > 35 kg/m^2^ und Alter < 60 Jahren sollte eine Verordnung von Metformin zur Senkung des Typ 2 Diabetes Progressionrisikos überlegt werden [36]. Aufgrund der Assoziation von längerer Einnahme von Metformin mit Vitamin B12 Mangel, sollte die Serumkonzentration von Vitamin B12 regelmäßig kontrolliert werden [36].

### Ansätze zur Prävention des Typ 1 Diabetes

Die Prävention des Typ 1 Diabetes kann auf 3 verschiedenen Ebenen stattfinden: (i) primäre Prävention (in der frühen Kindheit) vor Immunaktivität gegen die Betazelle, (ii) sekundäre Prävention bei noch bestehender Normoglykämie aber humoralen oder metabolischen Parametern mit hohem Risiko für die Entwicklung eines Diabetes und (iii) tertiäre Prävention mit dem Versuch der Verlängerung der Betazellfunktion bei bereits manifestem Typ 1 Diabetes. Etablierte Methoden sind derzeit noch nicht entwickelt, jedoch ist eine primäre Prävention durch Impfungen (GAD65, CVB1 basiert) oder mikrobiotainduzierte Immunregulation denkbar [64–66]. Einige internationale randomisierte multizentrische Studien zur primären Prävention von Typ 1 Diabetes waren nicht erfolgreich (DENIS, ENDIT, DIAMYD, DPT-1) [67]. Geringe orale Insulindosen konnten im einem RCT im Vergleich zu Plazebo die Entwicklung eines Typ 1 Diabetes nicht verhindern [68]. Eine laufende randomisiert kontrollierte Studie versucht dies nun bei Kleinkindern mit erhöhtem Risiko für Typ 1 Diabetes zu untersuchen [69]. Daten aus Finnland weisen darauf hin, dass Kinder mit erhöhtem Risiko für Typ 1 Diabetes, die in den ersten drei Lebensmonaten mit Kuhmilch ernährt werden, ein erhöhtes Diabetesrisiko aufweisen, wohingegen Stillen in den ersten vier Lebensmonaten einen protektiven Einfluss haben könnte [70, 71]. Desweitern zeigten zwei Studien (DAISY und BABYDIAB) einen Zusammenhang zwischen Diabetes-assoziierten Autoantikörpern und diätetischer Gluten-Aufnahme, sodass in den ersten drei Lebensmonaten Gluten-haltige Nahrungsmittel nicht gefüttert werden sollten [72, 73].

Eine sekundäre Prävention könnte aus Kombinationstherapien aus immunmodulatorischen, antiinflammatorischen und Glukosestoffwechsel-verbessernden Medikamenten bestehen [64]. Ein monoklonaler anti-CD3-Antikörper (Teplizumab) zeigte bei Angehörigen von Menschen mit Typ 1 Diabetes eine Progressionsverzögerung im Vergleich zu Plazebo [74]. Teplizumab ist jedoch derzeit nicht für die Prävention bei Menschen mit Typ 1 Diabetes zugelassen.

Zur tertiären Prävention, also zur Vermeidung der Progression eines bereits manifestierten Typ 1 Diabetes durch Erhalt der Betazellmasse oder durch Verlängerung der klinischen Remission (Honeymoon-Phase), ist ebenso keine Therapie zugelassen. Auch in dieser Indikation werden Immuntherapeutika untersucht [75]. Solange der Mechanismus der Entstehung von Typ 1 Diabetes nicht ausreichend verstanden ist und das kostspielige Screening mittels Diabetes-assoziierter Autoantikörper nicht durch günstigere Alternativen abgelöst wird, ist es schwierig gezielte und langfristig effektive Präventionsstrategien zu entwickeln und etablieren. Derzeit kann ein allgemeines Screening mittels Autoantikörpern nicht empfohlen werden, da keine zugelassenen Interventionsmöglichkeiten vorliegen [36]. Im Fall eines positiven Nachweises von Autoantikörpern wird allerdings empfohlen eine Beratung zu den Themen Diabetes, Symptome des Diabetes und Diabetische Ketoazidose durchzuführen [36].
